# Grey scale enhancement by a new self-made contrast agent in early cirrhotic stage of rabbit liver

**DOI:** 10.1186/1471-230X-7-32

**Published:** 2007-08-08

**Authors:** Li Zhang, Yun-You Duan, Ji-Kai Yin, Ji-Hong Cui, Yong Zhang, Tie-Sheng Cao

**Affiliations:** 1 Department of Ultrasound Diagnosis, Tangdu Hospital, Fourth Military Medical University, Xi'an, China; 2 Department of General Surgery, Tangdu Hospital, Fourth Military Medical University, Xi'an, China; 3 Department of Pathology, School of Basic Medicine, Fourth Military Medical University, Xi'an, China

## Abstract

**Background:**

The development of new ultrasound contrast agents (UCAs) has become one of the most promising fields in ultrasound medicine. This paper evaluates a new self-made contrast agent enhancement effect developed to study the fibrotic stages of the liver in perfusion models *in vivo*.

**Methods:**

We constructed experimental models of hepatic fibrosis involving five stages from F0 to F4 via administration of CCL_4 _(0.01 ml/kg BW) every 3 days for 3 months. The intrahepatic circulatory time of the contrast agent was analyzed via an image and Cine-loop display. Calculations of the perfusion-related parameters including the peak signal intensity (PSI) and peak signal intensity time (PIT) of the portal vein and parenchyma were obtained from an analysis of the time-acoustic intensity curve.

**Results:**

Hepatic artery to vein transmit time (HA-HVTT) was significantly shorter at F4 stage (mean 5.1 seconds) compared with those in other stages (mean 8.3 s, 7.5 s, 6.9 s, 6.6 s, P < 0.01). The average PSI difference of PV-parenchyma was 13.62 dB in F4 stage, demonstrating significant differences between F4 stage and other early stages (P < 0.001).

**Conclusion:**

These results indicate that the new self-made contrast agent is capable of indicating intrahepatic hemodynamic changes. HA-HVTT and the PSI difference of the microbubble perfusion in liver parenchyma and PV were considered to differentiate the degree of hepatic fibrosis between F4 and other early stages.

## Background

In the 1990s, with the introduction of the microbubbles, clinical and research applications for ultrasound were revolutionized. Microbubbles consist of a gas (air or perfluorocarbon) stabilized by a shell (denatured albumin, phospholipid, or surfactant or cyanoacrylate). Microbubbles are less than 10 μm in diameter so they can be intravenously injected to cross the lung bed producing a systemic enhancement. Special imaging software and the development of contrast microbubbles resulted in a rapid expansion of the use of microbubbles in ultrasound scanning. Microbubbles can be used as tracers and this has been exploited in liver scans to identify conditions characterised by arterio-venous shunting such as cirrhosis [1,2] and metastases [3,4] – where an early hepatic vein transit time indicates a haemodynamic abnormality. This approach parallels that of dynamic CT or MRI but ultrasound has the advantages of high spatial and temporal resolution.

Different contrast agents have different perfusion characteristics based on their components. In Lim AK's study, SonoVue and Levovist were compared in the circulation time. Results showed HVTT was significantly shorter with SonoVue than with Levovist; there was no significant difference in cardiopulmonary transit time. In Prof. Gao's earlier study, a new self-made lipid-coated contrast agent was used in healthy rabbits. The results proved that this new contrast agent is safe and has good perfusion characteristics in hepatic parenchyma and renal cortex at a minimal dose of 0.01 mL/kg body weight (BW). While the circulatory time and grey scale intensity of the microbubble in liver were not systematically studied.

Liver fibrosis has been shown to be a bidirectional process and a growing body of data from laboratory and clinical studies show that even advanced fibrosis and cirrhosis are potentially reversible [5]. Therefore, the need for an early and accurate diagnostic test for liver fibrosis is essential. Although liver biopsy is generally considered as the "gold standard" in grading liver fibrosis, the major limitations of liver biopsy and the absence of a suitable laboratory screening test for liver disease in clinical practice have resulted in the evaluation of imaging techniques for the detection of liver disease. In a recent study, the diagnostic accuracy of ultrasonography for cirrhosis was found to be 80% with discriminant analysis [6].

In order to further validate the safety and effectiveness of this new contrast agent in diffused liver disease, the liver fibrosis models were established in rabbits and the new self-made contrast agent applied and analyzed in this study.

## Methods

### Animal model

Twelve male New Zealand White rabbits weighing between 2.0 and 2.2 kg with healthy livers were used as subjects. The CCL_4 _was injected intra-abdominally in doses of 0.10 ml/kg BW every 3 days for 3 months. This regimen proved sufficient to produce an advanced stage of liver fibrosis. Rabbits were taken biopsied at 30, 40, 60 and 90 days from the commencement of the CCL_4 _injection. The samples extracted from the liver biopsy were fixed for histopathology.

After at least a 4 hours fast, the rabbits were anesthetized with "Xu Mian Xin" (Changchun Argo-Pastoral University, Jilin, China), at a dose of 1.5 mg/kg BW by IM injection. After the abdominal skin was shaved with an 8% Na_2_S solutions, the rabbits were placed in a supine position. Additionally, prior to the contrast agent examination, an ear vein passage was established.

### Staging of liver fibrosis

Representative blocks from each biopsied liver were embedded by routine paraffin-wax processing and serially sectioned at 5 um thickness. Afterwards, they were stained with routine dyes (hematoxylin and eosin) and Massion's trichrome for histopathological evaluation. These slides were used for determining the stages of fibrosis by a pathologist, using the five-point METAVIR scoring system (F0-F4) [7]. This scoring system is defined as follows: F0, no fibrosis; F1, portal fibrosis without septa; F2, portal fibrosis with rare septa; F3, numerous septa without cirrhosis; and F4, cirrhosis.

### Ultrasonographic examination

In order to minimize or avoid the effect of liver trauma brought on by the biopsy, the ultrasound examinations were delayed by two days after the pathological changes were verified by biopsy. All subjects were tested after at least a four hour fast. Harmonic studies were all performed with a ViVid 7 (Logic, America) using a 10 MHz probe. All examinations were performed with rabbits in the supine position. Both the depth of field and focal zone were kept constant during the examination. The right lobe of lthe iver containing the right portal vein, right hepatic vein and right hepatic artery were simultaneously scanned in a transverse section.

The new self-made contrast agent used in this study was generously presented by the Third Military Medical University. The exact ingredients of the contrast agent used in this article include liposome, consisting of two kinds of phospholipids and polyethylene glycol; certain media including glucose and de-ionized water; perfluoropropane, which were introduced into the suspension during sonication. The bubbles concentration was 7 × 10^9^/ml and the size distribution was between 2 to 10 μm. Approximately 90% of the microbubbles were less than 8 μm. The surface electric potential was -71.2 mV and the pH was 6.42. The manufacture procedure performed as previously described [8].

The self-made contrast agent (0.01 mL/kg BW), was applied in a randomized order into a marginal ear vein as a bolus followed by a 5 ml saline flush. Mechanical index (MI) was deceased to 0.24, an experimental value verified by Professor Gao et al. Scanning setting was for optimal visualization, with the gain, scanning depth, field of view and time gain control (TGC), were determined throughout the study. After these conditions were determined, the transducer was held in a fixed position during examination. Bubble perfusion images were obtained by Cine-loop. The delay between the time of first bubble appearance in the hepatic artery, portal vein and hepatic vein was defined as the arrival time for corresponding vessels. HA-HVTT was defined as the time interval from the arrival time of HA to the arrival time of HV. The time-acoustic intensity curve was analyzed automatically by the quantitative analysis software package carried by ViVid 7 after contrast enhancement ultrasound. PV- parenchyma PIT was defined as the time interval from the peak signal intensity of PV to the peak signal intensity of parenchyma, measured from the time-acoustic intensity curve. PV-parenchyma PSI was assessed by the time-acoustic intensity curve. All ultrasound examinations and data analyses were performed by investigators blinded to the pathological changes of liver.

### Statistical analysis

Data were expressed as Mean ± Standard deviation. The analysis of variance was performed by GLM-ANOVA and subsequent pairwise comparisons were assessed with Tukey's procedure accounting for both animal factor and stage factor and the fact that some animals contribute to only a few stages. Statistical significance was defined as P < 0.01.

## Results

**Figure 1:**
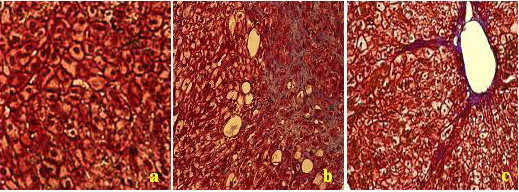
Histological changes in F2, F3 and F4 stage (× 200) using Massion's trichrome stain. (a) Increasing degeneration of liver cells, some fatty change of liver cells were observed in F2 stage; (b) Widespread changes in fatty cells and some fiber lesions (blue stained) in F3 stage; (c) Pseudo-lobule evident in F4 stage.

None of the rabbits in the 30-day treatment group died. Among the 40-day group, 3 rabbits died and by the 60th day, the death rate was 33% and by late on the 90th day, half of the animals had died from gastroenteric haemorrhagic necrosis to CCL4 and haemorrhage from the liver biopsy. None of the rabbits died from the administration of the contrast agent. According to the METAVIR scoring system, we had established 12 F0 and F1 stage models; 9 F2 stage models; 8 F3 stage models and 6 F4 stage models (Fig 1).

**Figure 2:**
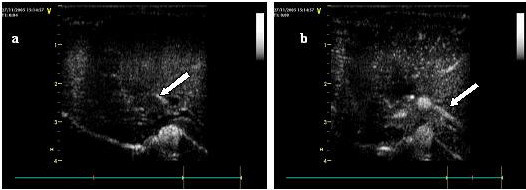
Illustration of the examination process. (a) 4 s after a bolus injection of the self-made contrast agent. First, the contrast agent arrived at the hepatic artery (arrow) 4 s after the injection. PV was only slightly enhanced. (b) 6 s after a bolus injection of the self-made contrast agent. The contrast agent then arrived at both the HA and PV (arrow).

**Figure 3:**
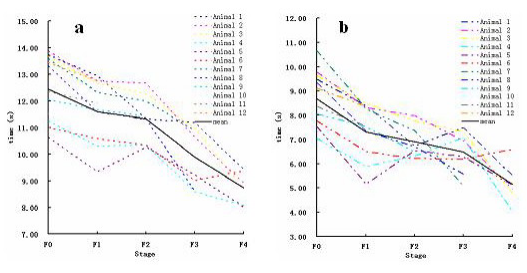
HVAT and HA-HVTT comparisons among all experimental animals at different stages. The general tendency is decent with the development of liver fibrosis both for HVAT (a) and HA-HVTT (b).

The circulatory times are summarized in Table 1. At 4 s after the bolus injection of the self-made contrast agent, the first microbubble appeared in hepatic artery and enhanced quickly (Fig. 2a), then the first microbubble arrived at the portal vein at 6 s after injection (Fig. 2b). HAAT, PVAT were not significantly different among five stages with the mean value of 4 s and 6 s respectively. HVAT varied at different stage. As shown in Fig. 3a, HVAT decreased gradually from F0 stage to F4 stage, but comparison between F4 and F3 stage showed no significant difference, neither did the comparison between F3 and F2 stage, F2 and F1 stage, F2 and F0 stage, F1 and F0 stage. A parallel reduction was obtained in HA-HVTT as shown in Fig. 3b, only in F4 stage in the rabbit liver HA-HVTT with a mean of 5.16 s differed significantly from other four stages (P < 0.01). Comparison between each set of two groups from F0 to F3 stage demonstrated no statistical differences for HA-HVTT. Results are listed in Table 2 showing that HA-HVTT is able to discriminate F4 stage and other early fibrotic stages.

**Figure 4:**
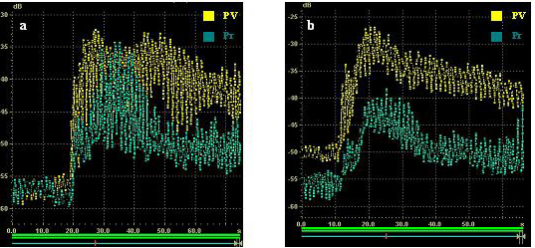
The time-acoustic intensity curve of a rabbit liver in F4 stage (a) and F0 stage (b).

**Figure 5:**
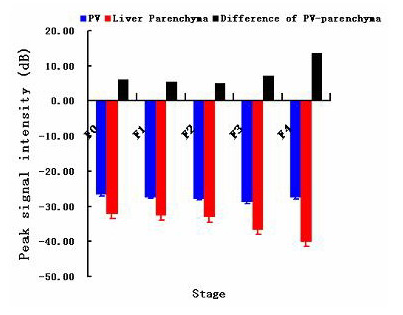
Average values for PSI of PV, parenchyma and difference of PV and parenchyma.

Time-acoustic intensity curves indicated the changes of intensity in the portal vein and liver parenchyma as the time period processed. Analysis of the profile of the time acoustic-intensity curve, showed a rapid-rising slope from the time of the bolus injection to the PSI and a gradual-descending slope after reaching PSI in both the portal vein and liver parenchyma as shown in Fig. 4. PIT measured was seen to be a little earlier in the portal vein than that in the parenchyma in every stage. Significant differences did not occur with PV-parenchyma PIT in the five stages. Fig. 5 showed the mean PSI parameters estimated for each stage. PSI of PV in F4 stage was -27.46 ± 0.22 dB, very similar to those in other stages (F0, -26.75 ± 1.09 dB; F1, -27.45 ± 0.35 dB; F2, -27.88 ± 1.35 dB; F3, -28.89 ± 2.73 dB; P > 0.01), while PSI of parenchyma decreased with the development of liver fibrosis. Parenchyma PSI both in F4 stage and F3 stage were clearly distinguishable from other four stages (P < 0.01). In F1 stage, the change of parenchyma PSI was incapable of indicating the difference from F0 stage and F2 stage. Interestingly, statistical analysis demonstrated that the PSI difference in PV and liver parenchyma between F4 stage and other four stages had statistical significance (P < 0.001). The statistical results of PSI difference by inter-group significance of paired comparison was not obtained among the F0, F1, F2 and F3 groups. Table 3 and Table 4 showed the results of PSI indicating PSI difference in PV and parenchyma is the indicative parameter.

**Table 1:** Circulatory time of the contrast agent in animal model at different stages ^a^

**Liver fibrosis stage**	**HAAT**	**PVAT**	**HVAT**	**HA-HVVT**	**PIT**
F0	3.78 ± 0.53	5.81 ± 0.16	12.54 ± 1.21	8.68 ± 1.13	1.13 ± 0.12
F1	4.28 ± 0.16	6.48 ± 0.22	11.59 ± 1.21	7.31 ± 1.17	0.94 ± 0.13
F2	4.40 ± 0.33	7.22 ± 0.32	11.33 ± 0.88	6.90 ± 0.64	1.47 ± 0.11
F3	3.39 ± 0.42	6.91 ± 0.44	9.90 ± 1.13	6.49 ± 0.86	0.82 ± 0.42
F4	3.58 ± 0.58	6.63 ± 0.43	8.74 ± 0.61	5.16 ± 0.85	1.16 ± 0.26

^a^Values are Mean ± SD

**Table 2:** Turkey's T test applied to the stages F0, F1, F2, F3 and F4 for HVAT and HA-HVTT parameters

Variables	Stages									
	
	F0/F1	F0/F2	F0/F3	F0/F4	F1/F2	F1/F3	F1/F4	F2/F3	F2/F4	F3/F4
HVAT	n.s.	n.s.	<0.01	<0.01	n.s.	<0.01	<0.01	n.s.	<0.01	n.s.
HA-HVVT	n.s.	n.s.	n.s.	<0.01	n.s.	n.s.	<0.01	n.s.	<0.01	<0.01

n.s.: non-significant

**Table 3:** PSI of the contrast agent in animal model at different stage ^a^

**Liver fibrosis stage**	**PSI of PV**	**PSI of parenchyma**	**PSI difference of PV and parenchyma**
F0	-26.75 ± 1.09	-32.24 ± 0.89	6.09 ± 1.54
F1	-27.45 ± 0.35	-32.71 ± 0.46	5.40 ± 0.53
F2	-27.88 ± 1.35	-33.19 ± 0.51	5.00 ± 1.36
F3	-28.89 ± 2.72	-37.34 ± 0.69	7.12 ± 1.07
F4	-27.46 ± 0.22	-41.08 ± 0.86	13.62 ± 0.80

^a^Values are Mean ± SD

**Table 4:** Turkey's T test applied to the stages F0, F1, F2, F3 and F4 for PV PSI, parenchyma PSI and PV-parenchyma PSI difference parameters

Variables	Stages									
	
	F0/F1	F0/F2	F0/F3	F0/F4	F1/F2	F1/F3	F1/F4	F2/F3	F2/F4	F3/F4
PV PSI	n.s.	n.s.	n.s.	n.s.	n.s.	n.s.	n.s.	n.s.	n.s.	n.s.
Parenchyma PSI	n.s.	<0.01	<0.01	<0.01	n.s.	<0.01	<0.01	<0.01	<0.01	<0.01
PSI difference	n.s.	n.s.	n.s.	<0.01	n.s.	n.s.	<0.01	n.s.	<0.01	<0.01

n.s.: non-significant

## Discussion

In this study, we have shown that at low MI harmonic real-time imaging, the new self-made contrast agent exhibited strong stability in the enhancement of hepatic parenchyma among different fibrotic stages of rabbit liver. Using real-time low-MI mode, the time-intensity curve could be obtained more accurately because bubbles are better preserved. As previously demonstrated, the perfusion course of contrast agents has the following properties: a parenchyma enhancement phase in the liver parenchyma following the enhancement of the blood pool and lasting for a relatively longer period, (also called late-phase). Our results showed that the self-made contrast agent reaches the hepatic artery 4 s after administration, and reaches the portal vein 2 s later. As noted in the previous study, grey scale enhancement could last more than 50 min from both visual assessment and quantitative statistical analysis [8]. In this study, we observed the parenchyma enhancement only for the first 5 minutes to assess peak signal intensity instead of the whole parenchyma enhancement period. The self-made contrast agent characteristics are demonstrated in the time acoustic-intensity curve profile.

HAAT and PVAT are very similar among the five fibrotic stages. With the development of fibrosis, HVAT is earlier and HA-HVTT shortened. In F4 stage, intrahepatic shunts reduce the transit time through the liver, since a significant amount of blood bypasses the liver sinusoids. In spite of the similar change tendency, HVAT is incapable to discriminate every stage from the earlier stage. HA-HVTT is significantly shorter than those of other early stages with mean of 5.13 s respectively. The tendency that HA-HVTT in liver cirrhosis is significant shorter than that in normal supports earlier studies [9,10].

The PSI in the portal vein is higher than that in the liver parenchyma at every assessment stage, supporting Li J's results [11]. As the fibrosis developed, a progressive reduction of parenchyma PSI was observed in the late phase, which is accordance with Kaneko T's studies with Levovist [2], but did not occur with PV PSI, accordingly the gap between PV and parenchyma PSI is increased. Statistical analysis show us that PV-parenchyma PSI difference is more suitable than parenchyma PSI in separating the stages of different degrees of fibrosis, which we found, interestingly, similar with the analysis of contrast agent transmit time.

A clear explanation of the reduction of parenchyma enhancement is difficult to establish. If the contrast microbubbles simply pool in the hepatic sinusoids, the anatomical changes of these vessels occurring in a cirrhotic liver, such as capillarization, could be considered as a possible explanation for PSI in PV always being higher than that in parenchyma and PSI in PV being less affected by the changes caused by liver fibrosis. The mean diameter of the microbubble is 3–5 μm, a little larger than that of the commercially available UCAs, such as SonoVue with a mean diameter of 2.5 μm [12], however, more than 80% of microbubbles are less than 8 μm. It is difficult for a larger microbubble to diffuse in hepatic sinusoids efficiently, especially when liver stiffness increases.

However, the site of this type of microbubble accumulation is unknown. We assumed that the hepatic enhancement phase after perfusion in blood-pool may be from uptake by the reticuloendothelial system in liver, where Kupffer cells play an important role [13,14]. But in other results from anatomical studies, the number of hepatic Kupffer cells does not differ significantly in normal subjects or in cirrhotic ones [15]. The observed changes could possibly be due, at least in part, to impaired functional capacity of the Kupffer cells. The post-vascular enhancement of Levovist lasts from several minutes to 1 hour, the new self-made contrast agent can enhance in hepatic parenchyma more than 50 min. In recent studies, Levovist was confirmed to be phagocytosed by Kupffer cells both in vitro and in vivo [16]. Considering the similarity of the long post-vascular enhancement, the reduction of parenchyma enhancement may due to the Kuffer cells. The exact reasons need to be further studied.

In this study, greater emphasis was placed on HA-HVTT and the differences in PV-parenchyma PSI, minimizing the error brought through administration time delay, thus making the evaluation parameters less subjective. The circulatory characteristics confirmed that the new self-made contrast agent has the potential to differentiate early liver cirrhosis from liver fibrosis. The new self-made contrast agent based on lipids provides more advantages than conventional ultrasound. It can reflect liver perfusion, especially in satisfactory enhancement of the parenchyma *in vivo *in the animal model. As a consequence of the tendency to accumulate in the hepatic parenchyma, this contrast agent can also be considered to be confined to the vessels. In other words, based on the prolonged enhancement in liver parenchyma, this new contrast agent has potential in further diagnostic and therapeutic application by carrying tumor markers or therapeutic drugs. Further large-scale studies are required to accurately assess sensitivity and specificity before the new contrast agent can be adopted for clinical practice.

## Conclusion

In summary, the new self-made contrast agent is safe when applied in liver fibrosis models *in vivo*. It effectively reflects liver haemodynamic changes and can also indicate early liver cirrhosis by highlighting HA-HVTT and PSI difference between the portal vein and parenchyma. This data is expected to be useful for further studies in ultrasound diagnosis of liver fibrosis using this new contrast agent.

## Abbreviations

PSI, peak signal intensity; HA-HVTT, hepatic artery to vein transmit time; PIT, peak signal intensity time; UCAs, ultrasound contrast agents; ROI, region-of interest ; MI, Mechanical index; TGC, time gain control; HAAT, hepatic artery arrival time; PVAT, portal vein arrival time; HVAT, hepatic vein arrival time

## Competing interests

The author(s) declare that they have no competing interests.

## Authors' contributions

LZ performed the ultrasound examination, construction of the animal model and data analysis. YYD designed the studies. JKY and TW performed the administration of contrast agents and rabbit liver biopsies. JHC performed the classification of liver fibrosis stage. YZ performed the data collection. TSC reviewed the data. All authors have given final approval of the version to be published.

## Pre-publication history

The pre-publication history for this paper can be accessed here:


